# Supplementary material for: Molecular and Biochemical Evidence of the Toxic Effects of Terbuthylazine and Malathion in Zebrafish
Source: Animals (Basel). 2023 Mar 11;13(6):1029. doi: 10.3390/ani13061029 (PMC10044699; doi:10.3390/ani13061029)
Supplement: Supplementary file 1 [file animals-13-01029-s001.zip › animals-2248392-supplementary.pdf]

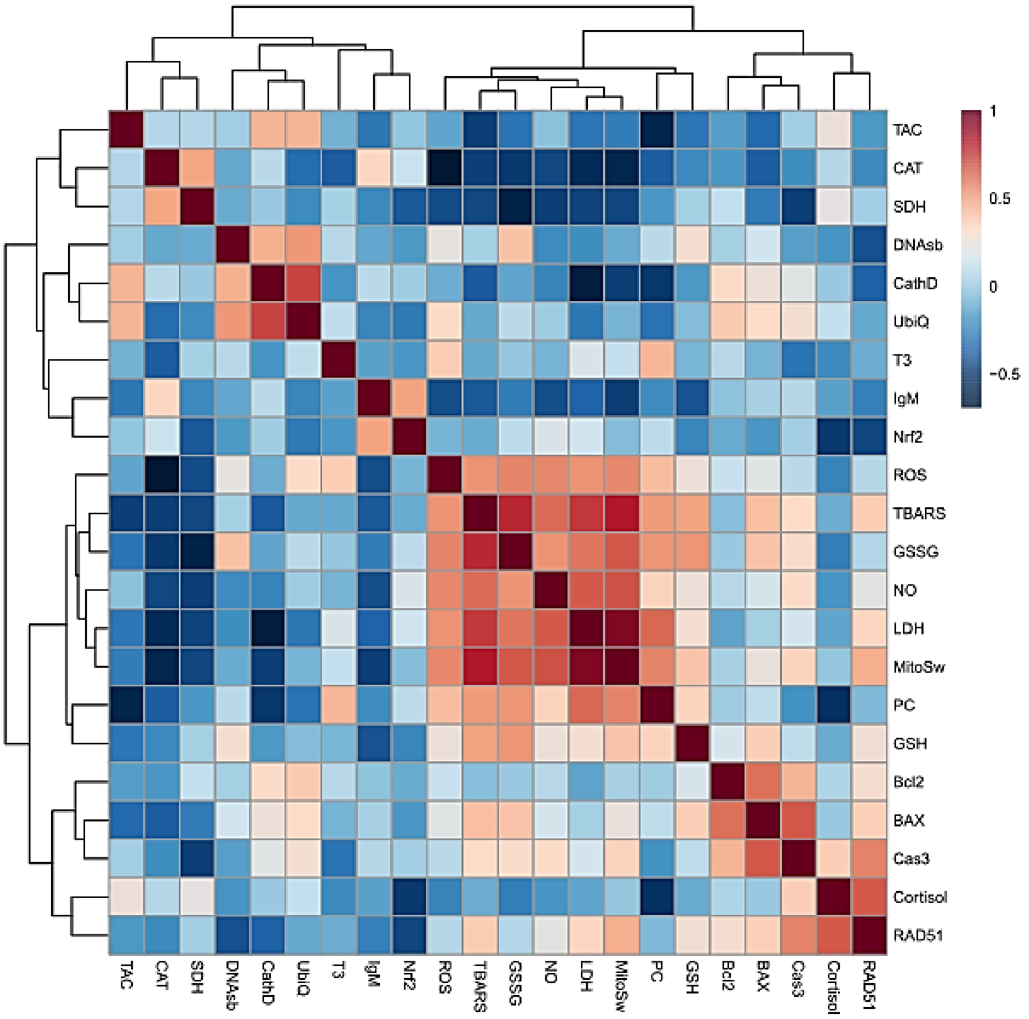

Figure S1: The correlation map of biological indices of zebrafish exposed to terbuthylazine and malathion.
